# Hydrogen reduction of spent lithium-ion battery cathode material for metal recovery: Mechanism and kinetics

**DOI:** 10.3389/fchem.2022.1019493

**Published:** 2022-09-26

**Authors:** Zhu Huang, Dawei Yu, Brian Makuza, Qinghua Tian, Xueyi Guo, Kun Zhang

**Affiliations:** ^1^ School of Metallurgy and Environment, Central South University, Changsha, China; ^2^ National and Regional Joint Engineering Research Center of Nonferrous Metal Resource Recycling, Changsha, China; ^3^ National WEEE Recycling Engineering Research Centre, Jingmen, China

**Keywords:** spent lithium-ion batteries, cathode material, hydrogen reduction, Ni-Co alloy, reaction mechanism, kinetics

## Abstract

Hydrogen reduction is becoming a promising method for recycling lithium-ion battery cathode materials. However, the reaction mechanism and kinetics during hydrogen reduction are unclear, requiring further investigation. Therefore, non-isothermal and isothermal reduction experiments were conducted to evaluate the temperature dependence of the hydrogen reduction kinetics using simultaneous thermogravimetric and differential thermal analysis equipped with mass spectrometry. XRD and SEM were used to characterize the reduction products to understand the underlying reduction mechanisms. The hydrogen reduction profile could be divided into three main stages: decomposition of cathode materials, reduction of the resultant nickel and cobalt oxides, and reduction of LiMnO_2_ and residual nickel and cobalt oxides. The hydrogen reduction rate increased with increasing temperature, and 800°C was the optimum temperature for separating the magnetic Ni-Co alloy from the non-magnetic manganese oxide particles. The apparent activation energy for the isothermal tests in the range of 500–700°C was 84.86 kJ/mol, and the rate-controlling step was the inward diffusion of H_2(g)_ within each particle. There was an downward progression of the reduction through the material bed for the isothermal tests in the range of 700–900°C, with an apparent activation energy of 51.82 kJ/mol.

## 1 Introduction

Lithium-ion batteries (LIBs) have become the preferred energy storage option in various fields such as transportation and aeronautics due to their excellent physical and chemical properties (Gao and Yang, 2010; Li et al., 2021). With the increased production of electric vehicles in recent years, there is a growing demand for LIBs (Yun et al., 2018). Meanwhile, the prices for battery metals continue to rise due to proliferating demand for these metals and the scarcity of mineral resources (Henckens et al., 2016; Mo and Jeon, 2018). For instance, the instability in the supply and price of lithium hinders the sustainable growth of LIBs production.

Generally, the lifespan of LIBs for electric vehicles is about 5–8 years (Zeng et al., 2012) which means that a large number of spent LIBs will be disposed of in the future (Richa et al., 2014; Yu et al., 2021; Chandra et al., 2022). Noteworthy, the content of valuable metals in spent LIBs is much higher than that in primary natural ores (Lombardo et al., 2019), and the extraction of the valuables metals from spent LIBs can be less complex than extraction from virgin ores (Huang et al., 2018). Moreover, recycling spent LIBs can result in energy savings and emission reductions (Yazicioglu and Tytgat, 2011; Abid Charef et al., 2017; Yang et al., 2020). According to Yazicioglu and Tytgat (2011), up to 70% of energy and emission reduction could be attained by recycling LiCoO_2_ batteries compared to metal extraction from primary ores. Therefore, recycling spent LIBs is crucial to the sustainable development of the lithium-ion battery and the electric vehicle industries.

The spent LIBs have been predominately recycled using pyrometallurgical or hydrometallurgical methods. The pyrometallurgical method involves processing the spent LIBs at elevated temperatures, and it is considered a dominant and mature process with fast reaction kinetics and a large processing capacity (Makuza et al., 2021b). Smelting has been widely applied as a pyrometallurgical recycling option at the laboratory and industrial scale to recycle spent LIBs. However, the smelting process suffers from the limited recovery of Li and Mn as they are lost in the slag, and the high-temperature requirements exacerbate recycling costs (Ren et al., 2016).

Recently, reduction roasting has been widely explored in a bid to enhance recycling efficiency. Reduction roasting offers the benefits of selective metals recovery, possible recovery of ignoble metals, and possible elimination of the usage of concentrated acids during the subsequent leaching process. Various solid reductants have been used during reduction roasting, such as reduction using a carbon source (carbothermic reduction) (Liu et al., 2019; Pindar and Dhawan, 2019; Zhang et al., 2020; Makuza et al., 2021a) and aluminum (thermite reduction) (Wang et al., 2019). Despite satisfactory leaching efficiencies, these solid reductants result in low purity products necessitating further processing steps to remove the residual reductants. For instance, the carbothermic reduction product requires an additional calcination step to remove residual carbon. Likewise, thermite reduction results in multi-stage leaching processes employing alkaline and acids to selectively leach out residual aluminum, which exacerbates the post-treatment costs and, ultimately, the recycling costs (Huang et al., 2022). Gaseous reductants such as NH_3_ (Xiao et al., 2021) and H_2_ (hydrogen reduction) (Kim et al., 2013; Liu et al., 2021) have also been explored, and the results were promising.

Recently, there has been mounting pressure to reduce emissions; thus, green recycling methods are more favorable (Jiang et al., 2021; Chen et al., 2022). Unlike reduction using NH_3_, which results in possible air pollution, hydrogen reduction curbs emissions by producing water vapor as the off-gas component and, most importantly, eliminates product contamination. Although hydrogen reduction of lithium-ion battery cathode materials is a promising approach, it is still in its infancy stage, with only a handful of publications on the recovery of valuable metals from the complex lithium-ion battery cathode material chemistries. Thus it is of paramount significance to undertake a systematic study to evaluate the possible reaction mechanisms and kinetics associated with hydrogen reduction of lithium-ion battery cathode materials. To the best of our knowledge, the kinetics of hydrogen reduction of cathode materials have not been studied. Thus, this work intends to conduct kinetic analysis to effectively understand the hydrogen reduction process and add to the existing knowledge.

In this work, the mechanism of hydrogen reduction of cathode materials of LIBs during the isothermal and the non-isothermal reduction process was studied. Then the kinetic analysis of the reduction process of the cathode materials was carried out by two iso-conversional methods (Kissinger-Akahira-Sunose (KAS) and Flynn-Wall-Ozawa (FWO) models) (Vyazovkin et al., 2011; Li et al., 2020). The model fitting method was used to study the hydrogen reduction behavior of the cathode powders under different temperatures for the isothermal experiments. The phase transition of the reduction product, the change of microscopic morphology, and the distribution of elements were also investigated to elucidate the reduction mechanism.

## 2 Materials and methods

### 2.1 Materials

Ni-rich Ni−Co−Mn (NCM) cathode material of LIBs was used for the study. The wet chemical analysis results show that the NCM cathode powders comprised 8.88 wt% Li, 49.07 wt% Ni, 6.71 wt% Co, 3.38 wt% Mn, and 0.14 wt% Al, the balance being oxygen. The presence of Al as a dopant in the NCM cathode material was to enhance its electrochemical performance. The SEM micrograph shows that the particles of the untreated cathode powders were irregularly sized and depicted a spherical morphology, and the main phase was indexed as Li_1.03_Ni_0.97_Co_0.1_Mn_0.1_O_2_ (Supplementary Figure S1).

### 2.2 Experimental

**FIGURE 1:**
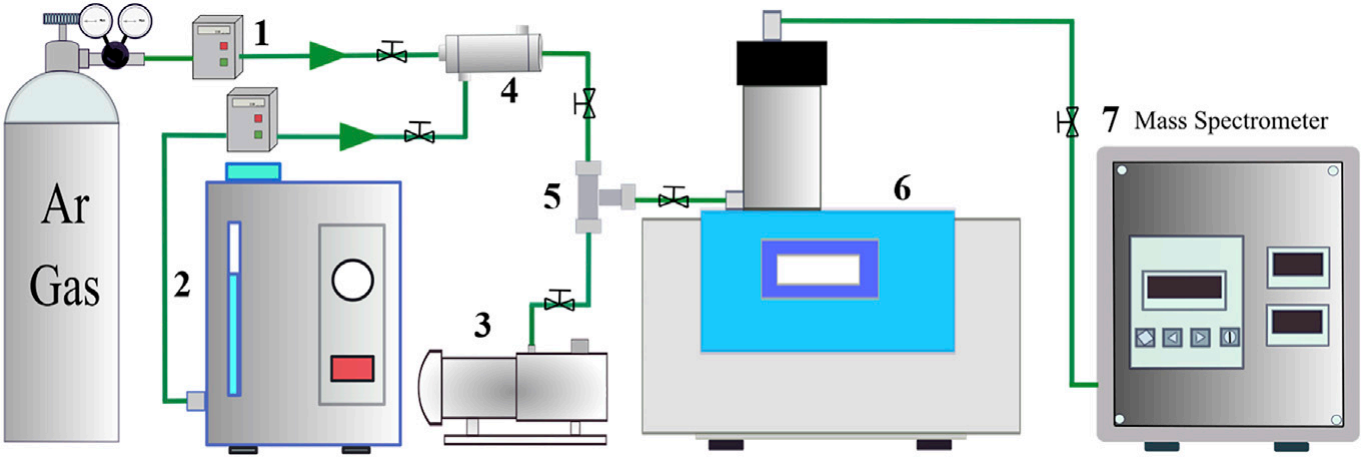
The experimental setup for the TG-DTA analyses. 1. Argon mass flow controller; 2. Hydrogen generator; 3. Vacuum pump; 4. Gas mixer; 5. Three-way valve; 6. TG-DTA; 7. Mass spectrometer.

The hydrogen reduction experiments were conducted using a simultaneous thermogravimetric and differential thermal analyzer (TG-DTA, HZT-4, Henven) coupled with a mass spectrometer (MS, PM-QMS100A, Jing-Protech) for the continuous measurement of gaseous H_2_O in the off-gas. The experimental setup is schematically shown in Figure 1. The hydrogen gas used during the experiments was produced using a hydrogen generator (SHC-300, Sykseth Hydrogen Energy) through the electrolysis of the KOH solution. The hydrogen generator was equipped with a water removal tube and a deoxygenation tube to remove the residual water and oxygen from the hydrogen gas stream. The purified hydrogen gas was mixed with high purity argon (5N) in different concentrations before being introduced into the TG-DTA.

Both the non-isothermal and isothermal reduction tests were conducted to study the reduction behavior of the LIB cathode powders. The cathode powders were heated from room temperature to 1,000°C using a predetermined heating rate for the non-isothermal tests. The gas mixture (H_2_ and Ar in various concentrations) was introduced at the onset of the heating process, and the flow rate was maintained at 100 ml/min during heating using mass flow controllers. H_2_ gas was turned off immediately after reaching the target temperature, and the samples obtained after cooling underwent various characterization methods to determine the reduction extent and mechanism.

A similar procedure was adopted for the isothermal tests. The differentiation was that for the isothermal tests, the samples were heated to the target temperature under an inert Ar atmosphere, and hydrogen gas was introduced for 3 h after reaching the target temperature. A heating rate of 10°C/min was used to reach the target temperature of 500°C, 600°C, 700°C, 800°C, and 900°C for the isothermal tests.

The sample mass change, heat flow, and the resultant gaseous H_2_O in the off-gas were continuously measured during the TG-DTA tests. A blank TG-DTA run was carried out for each experimental condition to ensure the accuracy of the analyses by eliminating the influence of factors such as the buoyancy effect.

### 2.3 Analytical methods

The chemical composition of the LIB cathode powders used for the experimental investigations was analyzed using an inductively coupled plasma optical emission spectrometry (ICP-OES, Optima 5300 DV, Perkin Elmer). The concentration of the gaseous H_2_O evolved during the non-isothermal and isothermal reduction tests were measured using a mass spectrometer (PM-QMS100A, Jing-Protech). The hydrogen reduction products were mounted in epoxy, ground, and polished to observe the morphology and chemistry of the products using a scanning electron microscope (SEM, MIRA3 LMH, TESCAN) equipped with an energy dispersive spectrometer (EDS, XMAX20, Oxford Company). Phase identification of the hydrogen reduction products was qualitatively analyzed using an X-ray diffractometer (XRD, D/Max-2500/PC).

## 3 Results and discussion

### 3.1 Non-isothermal reduction

#### 3.1.1 Hydrogen reduction characteristics

**FIGURE 2:**
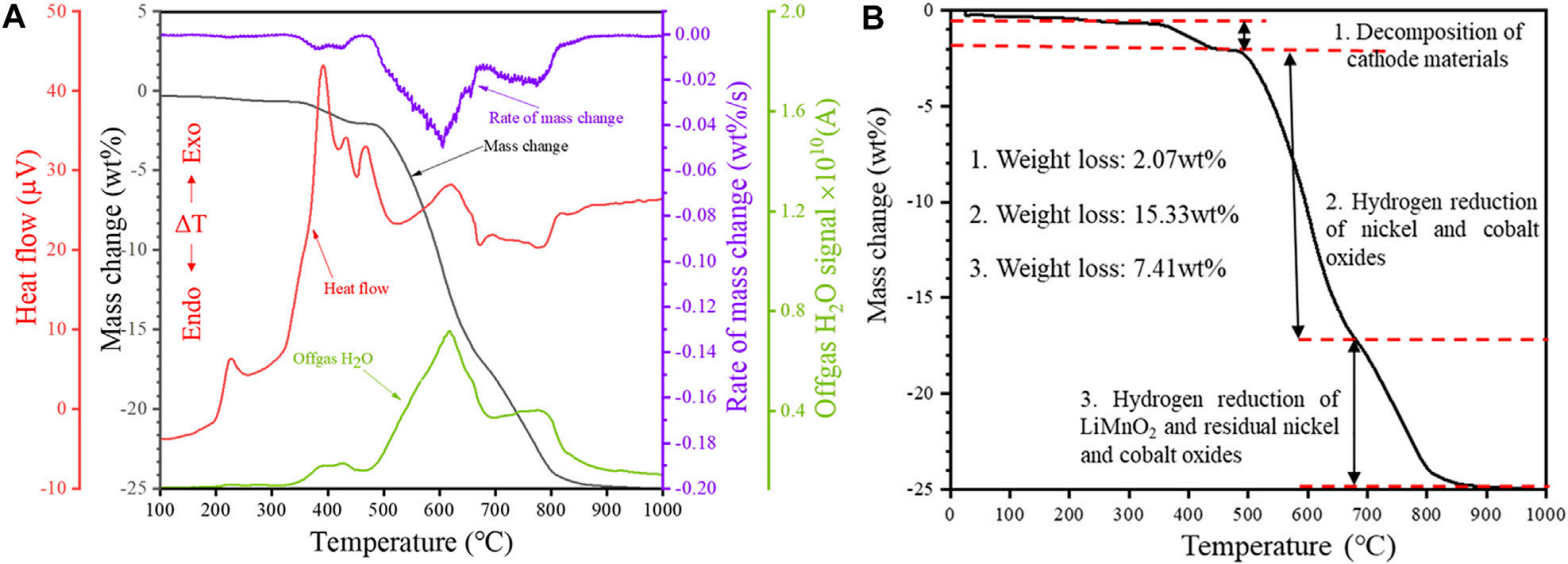
Non-isothermal reduction of the cathode powders: **(A)** TG-DTA-MS profile and **(B)** TG profile showing the possible reduction stages (Heating rate 20°C/min, H_2_ concentration 20 vol%).

Non-isothermal tests were carried out from room temperature to 1,000°C at a heating rate of 20°C/min using a 50 mg sample mass in order to investigate the reduction behavior of the cathode powders. The obtained results were plotted in Figure 2, and they comprise heat flow (μV), sample weight loss (wt%), rate of mass change (wt%/s), and signal of gaseous H_2_O concentration in the off-gas (A) measured by MS.

**FIGURE 3:**
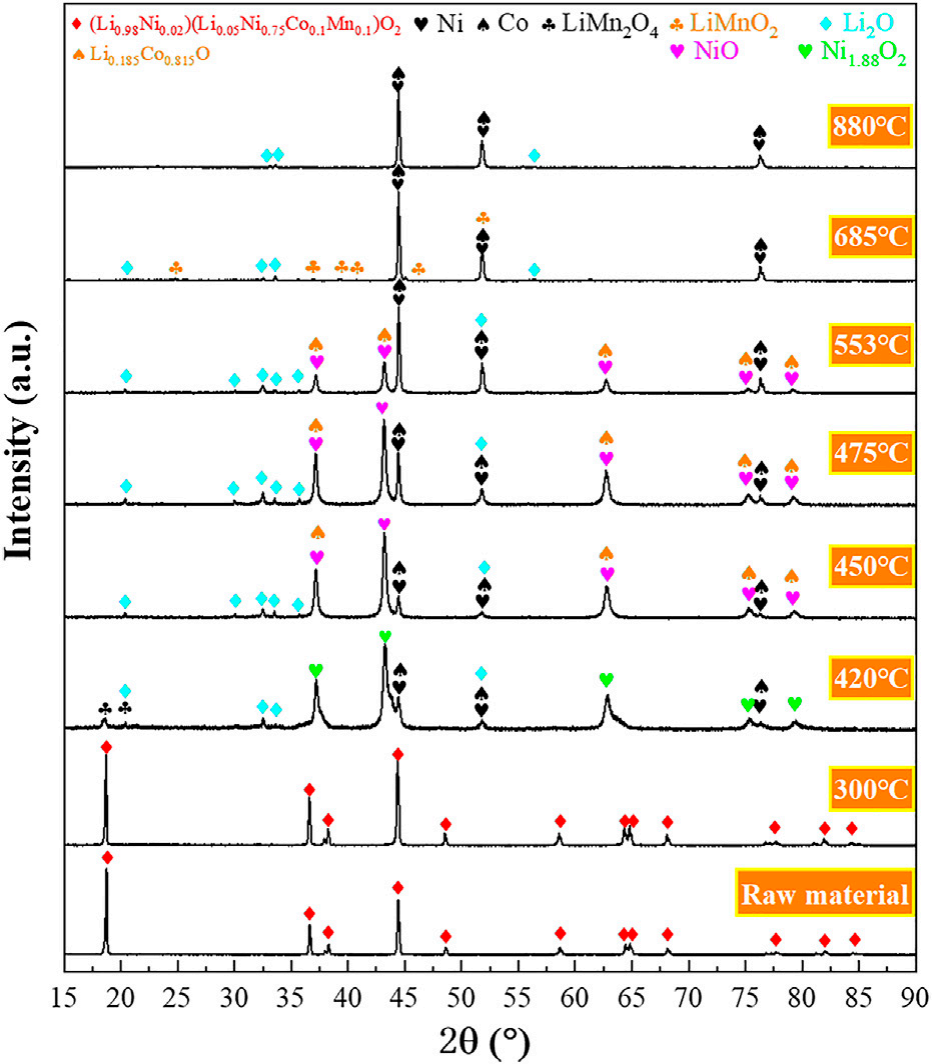
X-ray diffractograms of the reduction products obtained at intermediate reduction temperatures under non-isothermal reduction conditions (Heating rate 20 °C/min, Hydrogen concentration 20 vol% H_2_).

**FIGURE 4:**
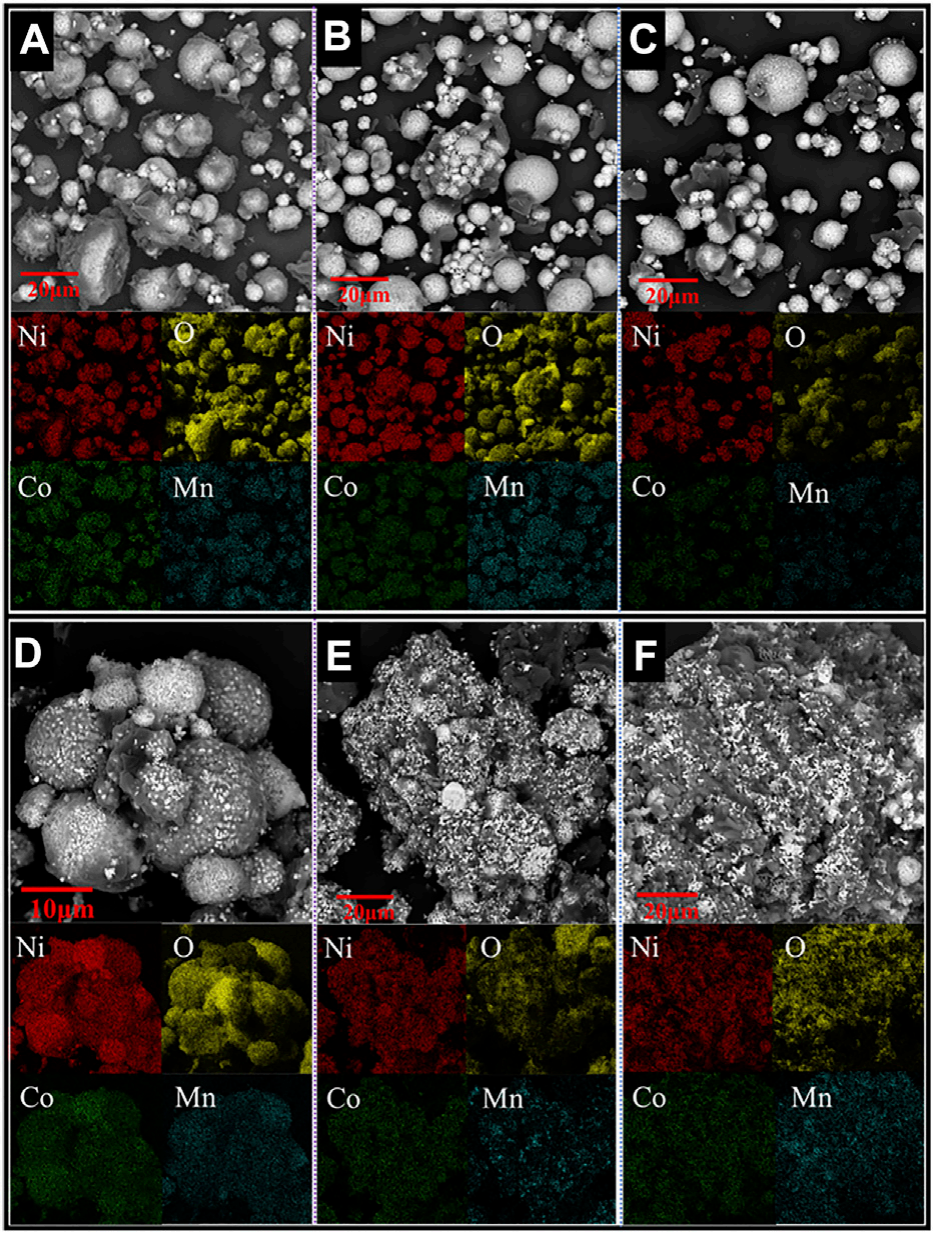
SEM and elemental mapping images of samples obtained at intermediate reduction temperatures of **(A)** 420°C, **(B)** 450°C, **(C)** 475°C, **(D)** 553°C, **(E)** 685°C, and **(F)** 880°C (Heating rate 20°C/min, H_2_ concentration 20 vol%).

The reduction products were collected at intermediate reduction temperatures and characterized to better understand the underlying reduction mechanism. The XRD and SEM analyses of the intermediate products are shown in Figures 3, 4, respectively. Based on the characterization of the phases present in the intermediate products, possible reactions for the hydrogen reduction of the cathode powders are depicted as reaction Eqs 1–5. Reaction Eqs 6–8 shows the possible subsequent reactions associated with intermediate compound formation, decomposition and reduction.

i) Reduction of the cathode material and resultant oxides

$${20Li}_{1.03}{Ni}_{0.97}{Co}_{0.1}{Mn}_{0.1}O_{2}+{\;3.562H}_{2\left(g\right)}=$$

$$9.8{Li}_{2}O+{\;10.319Ni}_{1.88}O_{2}+2CoO+Li{Mn}_{2}O_{4}+3.562H_{2}O_{\left(g\right)}$$ (1)

$${Ni}_{1.88}O_{2}+H_{2\left(g\right)}=NiO+H_{2}O_{\left(g\right)}$$ (2)

$$NiO+{\;H}_{2\left(g\right)\;}=Ni+H_{2}O_{\left(g\right)}$$ (3)

$${Co}_{3}O_{4\;}+{4H}_{2\left(g\right)}=3Co+{4H}_{2}O_{\left(g\right)}$$ (4)

$$CoO{+H}_{2\left(g\right)}=Co+{\;H}_{2}O_{\left(g\right)}$$ (5)

ii) Formation and reduction of intermediate compounds

$$10.811{Li}_{0.185}{Co}_{0.815}O+9.811H_{2\left(g\right)}={Li}_{2}O+8.811Co+{9.811H}_{2}O_{\left(g\right)}$$ (6)

$${Mn}_{2}O_{3}+{Li}_{2}O=2LiMnO_{2}$$ (7)

$$2LiMnO_{2}+H_{2\left(g\right)}={Li}_{2}O+2MnO+H_{2}O_{\left(g\right)}$$ (8)

It can be seen from Figure 2A that the profile of the rate of mass change (wt%/s) has a good correlation (near mirror symmetry) with that of the gaseous H_2_O evolution. This strongly suggests that the weight loss of the sample is closely related to gaseous H_2_O evolution. The total sample weight loss after the non-isothermal reduction test was 24.81 wt%. From the TG curve, it could be noted that higher temperatures favored the hydrogen reduction seen from the increasing weight loss with higher reduction temperatures. Based on the TG curve of the non-isothermal tests (Figure 2A), the hydrogen reduction profile of the cathode powders could be divided into three main stages, as shown in Figure 2B **.** Region 1 in the TG curve covers the temperature range from 25 to 475°C, and the sample weight loss in Stage 1 is 2.07 wt%. The first exothermic peak in the DTA curve appears at 195°C. The exothermic peak is accompanied by a slight weight loss and water signal between the temperature range of 195–300°C. Induced by the reducing atmosphere, the decomposition of the cathode material occurs in the temperature range of approximately 320–475°C, according to reaction Eq. 1, resulting in a significant H_2_O_(g)_ evolution. In this temperature range, multiple overlapping exothermic peaks can be observed on the DTA curve, suggesting a complex and multi-step thermal decomposition of the cathode material induced by H_2_.

The XRD results show that the cathode materials underwent no decomposition reaction at temperatures below 300°C (Figure 3). The X-ray diffractogram of the raw material and the product after reduction at 300°C are identical. Decomposition of the cathode powders was observed after reduction at 420°C, and the main phases detected in the reduction product were Li_2_O, Ni_1.88_O_2_, Ni, Co, and LiMn_2_O_4_, which aligns with the expected reduction products from reaction Eq. 1. However, a portion of Ni and Co oxides was further reduced to their metallic forms according to reactions Eqs 2–5. After being reduced, the cathode material maintained a spherical structure, and a dark phase appeared on the product surface (Figure 4).

According to the EDS elemental mapping, the dark phase mainly comprised oxygen, and the other elements (such as Ni, Co, Mn) were relatively low. Therefore, it could be speculated that the dark phase is Li_2_O (Li cannot be detected by EDS). It can be seen from Supplementary Figure S2 (EDS compositional analysis of the cross-section of particles present in the intermediate products) that the oxygen content in the regions closer to the particle surface is significantly lower than that closer to the center. This suggests the gas-solid reduction reactions followed the shrinking-core model involving the inward diffusion of H_2_ and outward diffusion of gaseous H_2_O.

It can be seen from Figure 3 that in the temperature range of 420–450 °C, the high-valent nickel oxide (Ni_1.88_O_2_) is reduced to NiO according to reaction Eq. 2, and the oxygen content in the particles was decreased, as shown in Figure S2a,b. The SEM micrographs shown in Supplementary Figure S2B, C and Supplementary Figure S3B, C show a further decrease in oxygen content with temperature increase from 450°C to 475°C, with the formation of more Ni/Co metallic phases (Figure 3).

Stage 2 covers the temperature range of 475–685°C, and the sample weight loss in Region 2 is 15.33 wt%, accounting for 61.79% of the total sample weight loss (Figure 2B). Likewise, the intensity of the peak corresponding to the Li_0.185_Co_0.815_O phase decreased significantly within 475–553°C. However, the intensity of the peak corresponding to nickel-cobalt alloy increased significantly. Thus, it could be speculated that the reduction of nickel oxides, cobalt oxides and complex compounds (such as Li_0.185_Co_0.815_O) mainly occurred in Stage 2, according to reactions Eqs 2–6.

It can be seen from Figure 4 that the product obtained at 553°C still retained the spherical shape. Figure 4D clearly shows that the particles were mainly composed of white nickel-cobalt alloy, and manganese was still uniformly distributed in the product. In the temperature range of 553–685°C, the mass change rate gradually increased and peaked at 606°C (Figure 2A). Likewise, the corresponding H_2_O_(g)_ signal also peaked at 606°C. Because more nickel and cobalt oxides are reduced by hydrogen, the original spherical structure of the particles gradually collapses, and the space between the particles decreases (Figure 4E). As a result, the H_2_ diffusion into the sample decreased, leading to a decreasing reduction rate when the temperature exceeded 606°C. The phase LiMnO_2_ is observed at 685°C (Figure 3), indicating that the reaction Eq. 7 forming LiMnO_2_ may have taken place at 475–685°C.

The third reaction stage corresponds to the temperature range of 685–880°C, and the sample weight loss in this stage is 7.41 wt%. It can be seen from Figure 3 that the peak corresponding to LiMnO_2_ disappeared at 880°C. Thus the LiMnO_2_ may have undergone reduction at 880 °C according to reaction Eq. 8. Based on the Mn concentration in the starting material, the weight loss corresponding to the reduction of LiMnO_2_ should not exceed 1 wt%; thus, the major weight loss in Stage 3 probably originated from the further reduction of nickel-cobalt oxides. The sample weight loss leveled off after the reduction temperature reached 880°C, indicating that the reduction process was almost completed before 880°C.

#### 3.1.2 Effect of hydrogen concentration and heating rate

**FIGURE 5:**
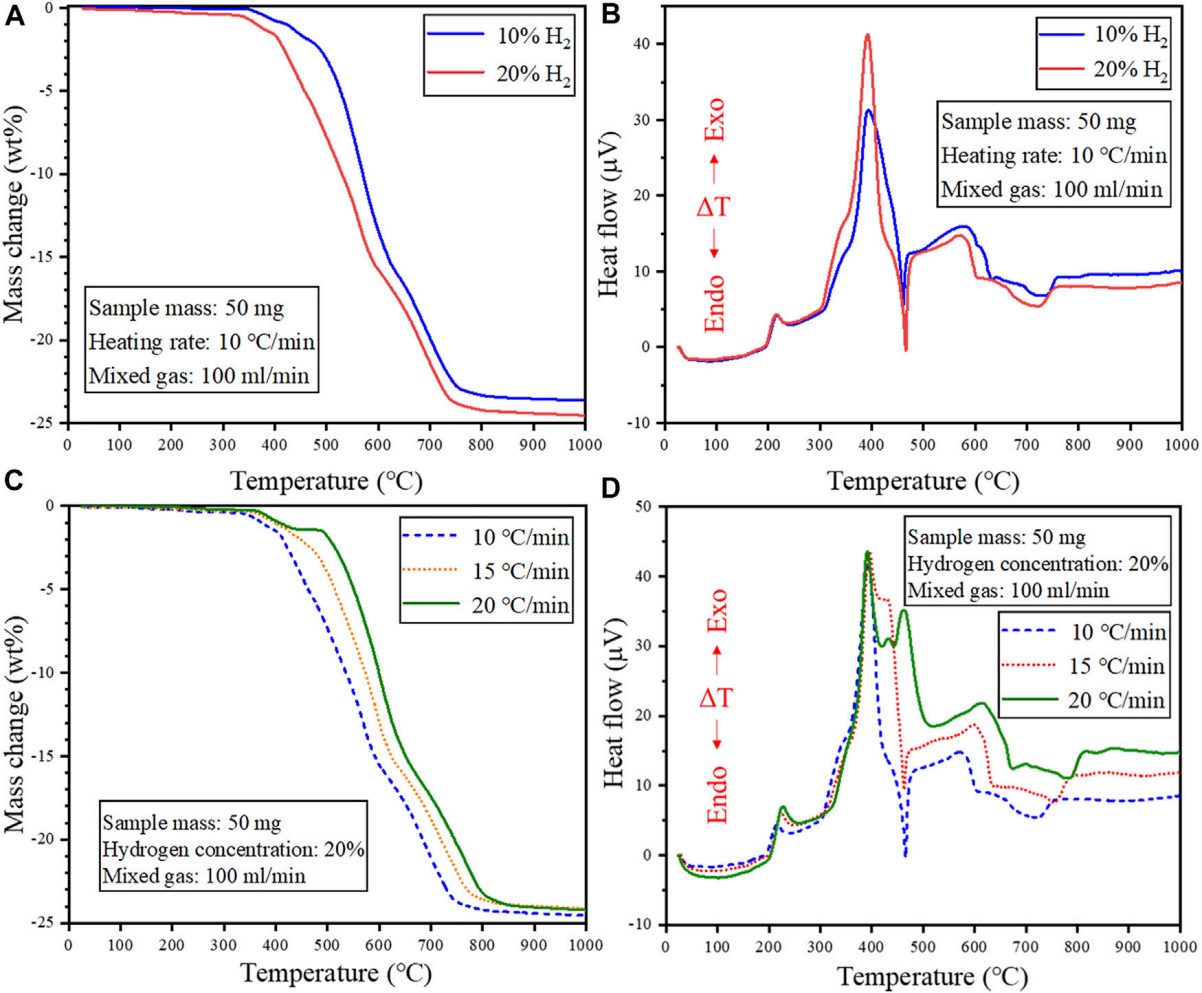
Effect of H_2_ concentration on the reduction behavior of cathode powders under non-isothermal reduction conditions **(A)** TG and **(B)** DTA curve. Effect of heating rate on the reduction behavior of cathode powders under non-isothermal reduction conditions **(C)** TG and **(D)** DTA curve.

The effect of hydrogen concentration and heating rate on the reduction behavior of cathode powders was investigated under non-isothermal reduction conditions, and the results are shown in Figure 5.

Figure 5A shows that increasing the H_2_ concentration shifted the TG curve to the low-temperature region, implying that higher H_2_ gas concentration could promote the reduction of the cathode powders, facilitating low-temperature recycling. However, increasing the heating rate shifted the TG curve to the high-temperature region, which is attributed to the shorter reaction time using fast heating rates, which limited the extent of the reduction reactions (Figure 5C). The DTA curve in Figure 5D shows the appearance of more peaks under fast heating rates, and this is attributed to the complex and multiple reactions coinciding under fast heating rates. This is because the reactions anticipated to occur at a certain temperature might be completed at higher temperatures due to limited reaction time under fast heating rates. Thus, a slower heating rate might be beneficial in ensuring the sufficient progression of the reduction reactions in each temperature region. In particular, an endothermic peak appears at about 470°C, when using heating rate of 10 and 15°C/min, while an exothermic peak was obtained at this temperature for 20°C/min. As discussed earlier, multiple overlapping exothermic peaks are observed in the temperature range of 320–475°C on the DTA curve at a heating rate of 20°C/min, suggesting a complex and multi-step thermal decomposition of the cathode material induced by H_2_. The presence of the endothermic peak at slower heating rates means the involvement of at least one endothermic reaction in the multi-step decomposition sequence. The disappearance of this endothermic peak at 20°C/min was possibly because it was completely canceled by the presence exothermic reactions. Therefore, it can be seen that the reaction mechanisms are strongly influenced by the heating rate.

### 3.2 Isothermal reduction

#### 3.2.1 Analysis of hydrogen reduction

**FIGURE 6:**
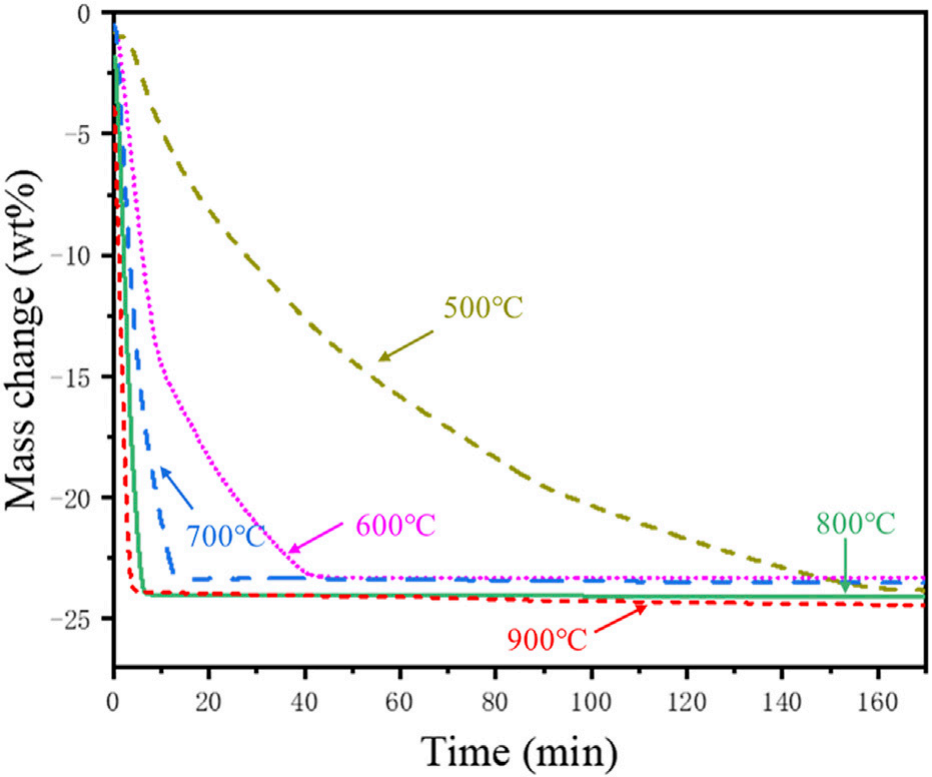
Mass change (wt%) during the isothermal reduction of the cathode powders.

Isothermal reduction tests were conducted to determine the influence of temperature on the reduction of the cathode powders. For the isothermal reduction tests, a sample mass of 46.8 mg, a heating rate of 10°C/min, and mixed gas flow rate of 100 ml/min (20 vol% H_2_) were used for the experimental investigations. The TG curves for the isothermal reduction tests are shown in Figure 6.

Figure 6 shows that higher reduction temperatures resulted in a faster and more pronounced reduction of the cathode powders with the gaseous hydrogen. The reduction rate at 500°C was slow, resulting in a gradual weight loss with a prolonged reduction. With the increase of the reduction temperature, the reduction rate accelerated, and the total reduction time decreased. The reaction was complete within 5 min of reduction at 900 °C, whereas the reaction was still ongoing after reduction at 500°C for 160 min. The total weight loss after reduction for 3 h at 500°C and 900°C was 23.32 wt% and 24.46 wt%, respectively. The SEM and elemental mapping images of the reduction products obtained after isothermal reduction at different temperatures are shown in Supplementary Figures S4, S5, and the corresponding X-ray diffractograms are shown in Supplementary Figure S6.

As shown in Supplementary Figures S4, S5, the particles after isothermal reduction at 500°C maintained a spherical structure similar to that of the particles obtained after non-isothermal reduction (Figures 4A–C). The spherical structure collapsed after isothermal reduction at 600°C due to the growth of alloy particles, and Mn became more dispersed. The reduction product at 700°C showed the formation of larger nickel-cobalt alloy particles, and the mapping of Mn indicated the formation of larger manganese oxide particles. Connected alloy particles resembling a dendrite structure could be observed at 800°C, which may have resulted from alloy sintering and growth (Supplementary Figure S5).

The oxygen elemental mapping of the particles is not entirely consistent with the distribution of manganese (Supplementary Figure S5), suggesting the presence of Li_2_O (Eq. 1). It could be speculated from the XRD phase analysis (Supplementary Figure S6) that the dark substance covering the surface of the alloy particles is Li_2_O. The reduction product obtained at 800°C underwent water leaching for 1 h with ultra-pure water. The SEM results of the recovered water leaching residue are shown in Supplementary Figure S7. The black deposits observed on the surface of the Ni-Co alloy in the SEM micrograph of the reduction product obtained at 800°C (Supplementary Figure S5E) could not be observed in the water leaching residue (Supplementary Figure S7). In addition, the oxygen distribution on the surface of the water leaching residue is consistent with manganese, proving that the dark phase is Li_2_O.

EDS point analysis was conducted on the magnetic nickel-cobalt alloy particles and non-magnetic oxide particles to explore the changes in elemental composition in the reduction product, and the results are shown in Supplementary Figure S8. Supplementary Figure S8A shows that higher reduction temperatures would favor the subsequent magnetic separation of the magnetic Ni-Co alloy particles from the non-magnetic manganese oxides. The Mn content in the magnetic Ni-Co alloy particles was much higher at 500°C and 600°C, and it gradually dropped with increasing reduction temperature and reached a minimum at 800°C. It can be seen from Supplementary Figure S8B that the content of Ni and Co in the non-magnetic oxide particles gradually decreased with increasing temperature as the residual Ni and Co oxides were reduced to form magnetic Ni-Co alloy. The average nickel content in the oxide particles was much higher at 500°C and 600°C.

#### 3.2.2 Effect of mixed gas flow rate and hydrogen concentration

**FIGURE 7:**
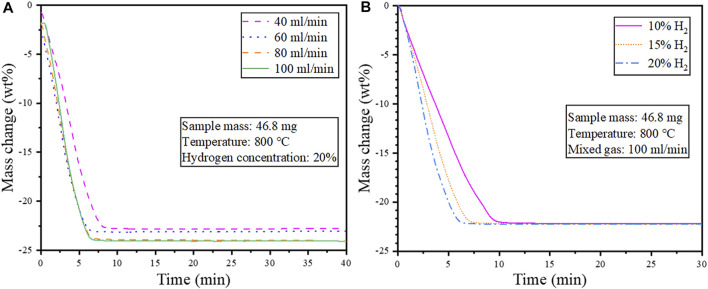
Isothermal reduction at 800°C **(A)** Effect of gas flow rate (heating rate 20°C/min); **(B)** Effect of H_2_ concentration on the reduction behavior of cathode powders.

Isothermal reduction tests were conducted to determine the effect of the mixed gas flow rate and hydrogen concentration on the reduction behavior of the cathode powders. A sample mass of 46.8 mg and a reduction temperature of 800 °C were used for the isothermal tests, and the result is shown in Figure 7.

As seen in Figure 7A, a lower flow rate of the mixed gas resulted in limited reduction and relatively low weight loss attributed to decreased diffusion and mass transfer. Hence a mixed gas flow rate of 100 ml/min was selected as the optimum condition for the subsequent tests on the effect of hydrogen concentration. Figure 7B shows that increasing the hydrogen concentration from 10–20 vol% increased the reduction speed. However, the weight loss remained unchanged with prolonged reduction.

#### 3.2.3 Effect of sample mass

**FIGURE 8:**
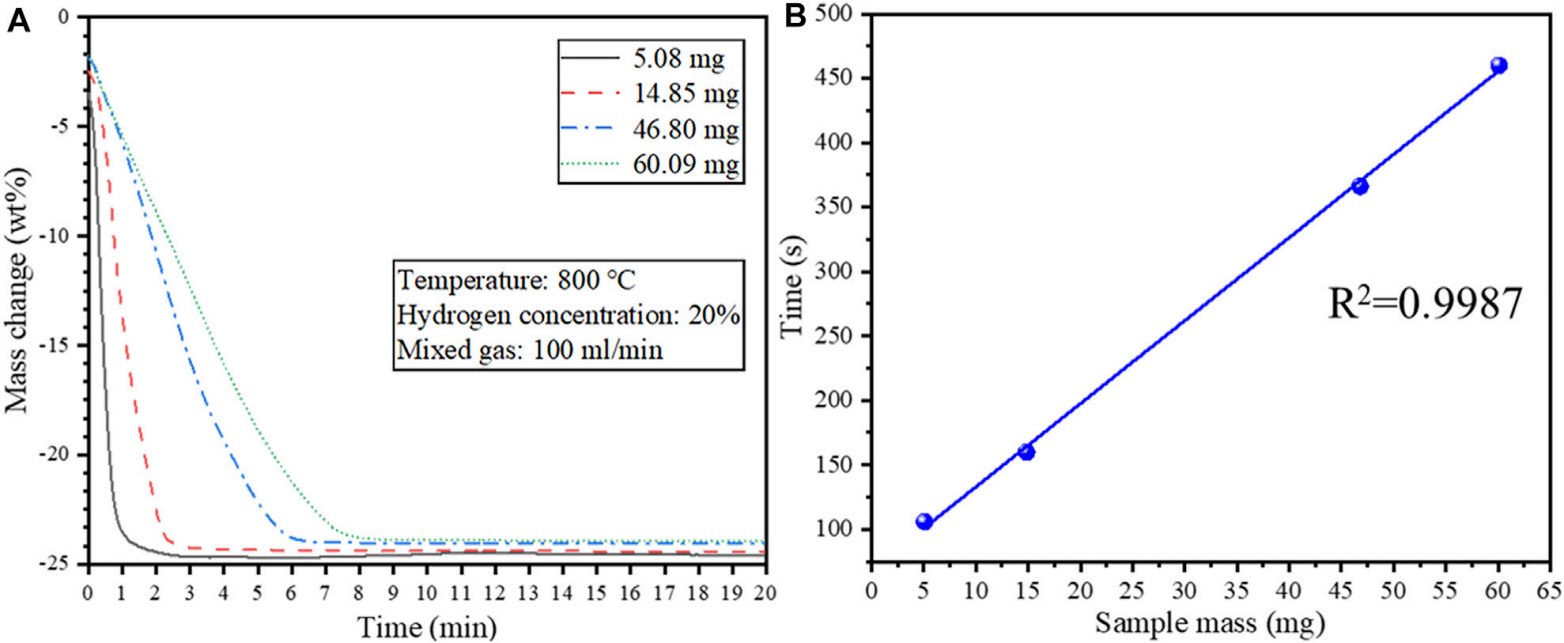
**(A)** Effect of sample mass on the isothermal reduction behavior of the cathode material at 800°C; **(B)** The relationship between the time required for complete reduction and the starting sample mass.

Isothermal reduction tests were conducted at 800°C to determine the effect of the sample mass on the reduction behavior of the cathode powders. Since the crucible size used in the experiments is fixed, changing the sample mass is equivalent to changing the material bed thickness. The TG curves under isothermal reduction as a function of sample mass are shown in Figure 8A. A 45° tangent was drawn at each TG curve in Figure 8A near the reaction endpoint, and the time at the intersection of the tangent line and the TG curve was defined as the reaction termination time. The corresponding relationship between the time when the reaction reaches the endpoint and the sample mass is shown in Figure 8B.

It can be seen from Figure 8A that the reaction was completed much faster when using a smaller sample mass attributed to a shorter diffusion path through the material bed. Noteworthy, the time for the reaction to complete has a linear relationship with the sample mass (*R* ^2^ = 0.9987) (Figure 8B). Therefore, the average reduction rate is constant even though the sample mass differed.

### 3.3 Kinetic analysis

#### 3.3.1 The KAS model and the FWO model

Hydrogen reduction of cathode materials is a relatively complex process involving multiple reactions. This section uses kinetic analysis to gain a better understanding of the hydrogen reduction process and to deduce the rate-controlling steps. The model-free method was employed first because it can obtain the apparent activation energy “Ea” without the need to establish a kinetic model for the process. Two representative iso-conversional methods, the Kissinger-Akahira-Sunose (KAS) model and Flynn-Wall-Ozawa (FWO) model, were then employed to analyze the reduction kinetics of cathode powders during non-isothermal reduction (Vyazovkin et al., 2011; Li et al., 2020).

The kinetic equations are derived as follows (Vyazovkin et al., 2011; Li et al., 2020):

$$\frac{d\alpha }{dt}=k\left(T\right)∙f\left(\alpha \right)$$ (9)

$$\alpha =\frac{m_{0}-m_{t}}{m_{0}-m_{\infty }}$$ (10)

$$k\left(T\right)=A∙e^{-\frac{E_{a}}{RT}}$$ (11)

$$\beta =\frac{dT}{dt}$$ (12)

In the formula, “α” represents the conversion rate of the sample, “t” is the reduction time (s), and “k(T)” represents the reaction rate constant with respect to temperature “T”, which can be expressed by the Arrhenius equation, denoted as Eq. 11, “ f(α) ” is the differential formula of the kinetic function, “m_0_” is the initial mass of the sample, “m_t_” is the mass of the sample at a certain time, and “m_∞_” is the mass of the sample when the reaction is over, “A” is the pre-exponential factor, “Ea” is the apparent activation energy of the reaction (kJ/mol), “R” is the ideal gas constant (8.314J/(mol·K)), and “β” is the constant heating rate.

$$\beta ∙\frac{d\alpha }{dT}=A∙f\left(\alpha \right)∙e^{-\frac{E_{a}}{RT}}$$ (13)

$$g\left(\alpha \right)=\int_{0}^{\alpha }\frac{d\alpha }{f\left(\alpha \right)}\approx \frac{A}{\beta }∙\int_{0}^{T}e^{-\frac{E_{a}}{RT}}∙dT=\frac{AE_{a}}{\beta R}P\left(y\right)$$ (14)

$$y=-\frac{E_{a}}{RT}$$ (15)

$$P\left(y\right)=-\int_{\infty }^{y}\frac{e^{-y}}{y^{2}}dy$$ (16)

$$\ln\left(\frac{\beta }{T^{2}}\right)=\ln\left(\frac{AE_{a}}{Rg\left(\alpha \right)}\right)-\frac{E_{a}}{RT}$$ (17)

**FIGURE 9:**
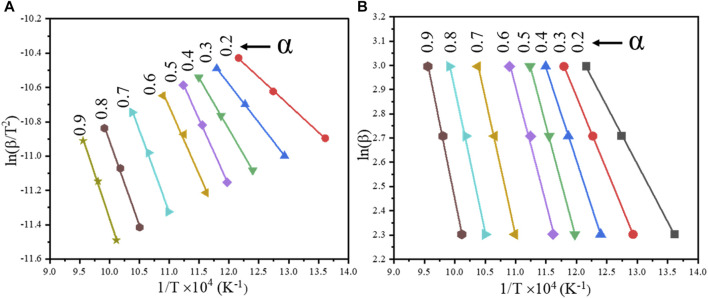
Linear fitting curves of the cathode materials at different conversion rates by iso-conversional methods: **(A)** KAS model; **(B)** FWO model.

Under specific conversion rates “α”, by plotting “ln (β/T^2^)” and “1/T” at different heating rates, the slope of the straight line at different conversion rates can be calculated to obtain the apparent activation energy “Ea” of the reaction, the result of which is shown in Figure 9A.

In the FWO model, the expression of P(y) is shown in Eq. 18, and the final form of the FWO model equation can be expressed as Eq. 19 (Li et al., 2020).

ln⁡P(y)=−5.331−1.052y (18)

$$\ln\left(\beta \right)=\ln\left(\frac{AE_{a}}{Rg\left(\alpha \right)}\right)-5.331-1.052\frac{E_{a}}{RT}$$ (19)

Under specific conversion rates “α”, by plotting “ln(β)” and “1/T” at different heating rates, the slope of the straight line can be calculated to obtain the apparent activation energy “Ea” of the reaction. The result is shown in Figure 9B.

It can be seen from Figure 9A that the slope of the fitted straight line under different conversion rates varies with the change in the conversion rate. The apparent activation energy under different conversion rates *α* was calculated by the slope of the fitted straight line. The calculated results are shown in Table 1.

**TABLE 1:** Kinetic parameters at different conversion rates *α* (KAS and FWO model).

Conversion rate (α)	KAS model		FWO model	
	E_a_/(kJ/mol)	*R* ^2^×10^2^	E_a_/(kJ/mol)	*R* ^2^×10^2^
0.2	26.71	99.92	37.64	99.93
0.3	37.27	99.98	48.21	99.99
0.4	49.74	99.99	60.51	99.99
0.5	64.13	99.90	74.57	99.95
0.6	65.30	97.95	76.12	98.69
0.7	79.48	99.48	90.35	99.66
0.8	81.65	99.38	93.10	99.59
0.9	86.64	99.56	98.43	99.72

As seen in Table 1, the apparent activation energy at the conversion rate of 0.2 is relatively low, and the sample particles maintained a spherical structure (Figure 4). With the progression of the reduction, the spherical structure of the sample particles gradually collapsed, and the activation energy increased steadily. The activation energy increased significantly when the conversion rate *α* was between 0.3 and 0.6, and the collapse of the sample particles would have reduced the reduction rate. Due to the collapse of the spherical structure, the alloy particles formed by the reduction reaction become compact and agglomerated (Figure 4). In addition, part of the unreacted nickel and cobalt oxides are wrapped by the alloy, which slows the diffusion rate of hydrogen into the alloy. Therefore, the activation energy increased significantly when the conversion rate *α* was in the range of 0.6–0.7. Compared with the KAS model, the *R* ^2^ of the FWO model is closer to 1 at a different conversion rate *α*, indicating that the FWO model is more consistent with this experiment.

#### 3.3.2 Model fitting

**FIGURE 10:**
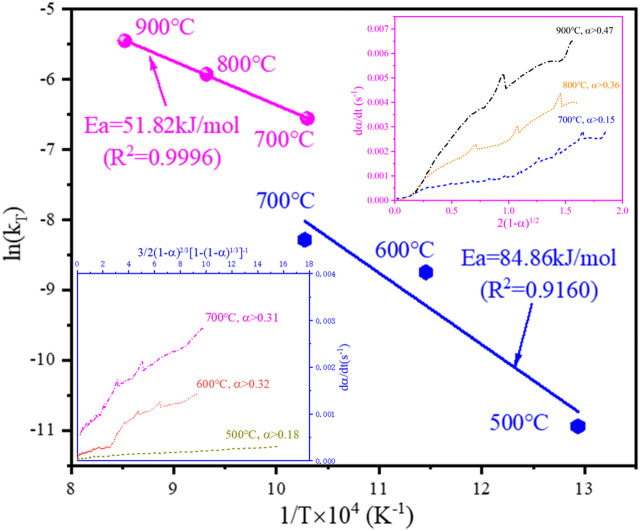
The Arrhenius plot for the isothermal reduction tests from 500°C to 900°C and the linear relationship between the rate of reaction and the differential form of the 3D diffusion model and R2 contracting area model.

In order to determine the rate-controlling step for the isothermal reduction tests between 500°C and 900 °C, the model fitting method was adopted. By analysis using the model fitting method (Figure 10), it can be concluded that in the temperature range of 500–700°C, the reduction process conforms to the 3D diffusion model (Khawam and Flanagan, 2006), and the differential expression of *f(α)* is shown in Eq. 20.

$$f\left(\alpha \right)=\frac{3}{2}{\left(1-\alpha \right)}^{\frac{2}{3}}{\left[1-{\left(1-\alpha \right)}^{\frac{1}{3}}\right]}^{-1}$$ (20)

Whereas in the range of 700–900°C, the reduction process conforms to the R2 contracting area model (Khawam and Flanagan, 2006), and the differential expression of *f(α)* is shown in Eq. 21.

$$f\left(\alpha \right)=2{\left(1-\alpha \right)}^{\frac{1}{2}}$$ (21)

In the above two kinetic models, *f(α)* and dα/dt have a good linear relationship, and the apparent activation energy of the reduction can be calculated from Eq. 22.

$$\ln\left(k_{T}\right)=lnA-\frac{E_{a}}{RT}$$ (22)

From Supplementary Figure S4B, C, it can be seen that the reduction product remained spherical at 500°C, and the spherical structure of the reduction product collapsed at 600°C. Therefore, when the conversion rate *α* is small, the reaction is mainly controlled by hydrogen diffusion into the material bed. As the oxides in the outer layer of each spherical particle are reduced, the reaction process conforms to the 3D diffusion model (Supplementary Figure S9A). The hydrogen gas then diffused through the product layer of each particle to react with the nickel and cobalt oxides inside the particle, so the reaction was controlled by the inward diffusion of hydrogen within the particle. As seen in Figure 10, the transition of the rate-controlling step from 3D diffusion to the R2 contracting area took place at 700°C, as the reaction process conforms to the R2 contracting area model when the conversion rate is greater than 0.15, and the reaction also conforms to the 3D diffusion model when the conversion rate is greater than 0.31 (Figure 10). Within 700–900°C, the reaction conforms to the R2 contracting area model (Supplementary Figure S9B). The chemical reaction rate was fast, and the hydrogen was rapidly consumed in the process of diffusion into the material bed. Thus the top surface of the material bed was initially reduced by hydrogen at a low conversion rate *α*, and it can be seen from Figure 10 that it did not conform to the R2 contracting area model at a low conversion rate (e.g., *α* ≤ 0.36 at 800°C). With the progression of the reaction, the top surface of the material bed inside the crucible became completely reduced, and the reaction front continued to progress downwards to reach the deeper unreacted material bed, and the reaction conformed to the R2 contracting area model (Supplementary Figure S9B). The effect of sample mass during isothermal reduction of the cathode powders at 800°C (Figure 8) further demonstrates the downward progression of the reaction front inside the material bed. It can be seen from the calculation that the apparent activation energy of the hydrogen reduction is 84.86 kJ/mol in the temperature range of 500–700°C, and it dropped to 51.82 kJ/mol in the temperature range of 700–900 °C.

## 4 Conclusion

The reaction mechanism and kinetics of the cathode powders of lithium-ion batteries during hydrogen reduction were investigated using TG-DTA-MS. The reduction products were analyzed using SEM-EDS and XRD. The results from the non-isothermal tests show that hydrogen reduction is mainly divided into three main reaction stages: The cathode material firstly decomposed under the reducing atmosphere in the temperature range of 195–475°C. Afterward, the reduction of nickel and cobalt oxides mainly occurred in the temperature range of 475–685°C, which resulted in the collapse of the spherical structure of cathode particles. Finally, the reduction of LiMnO_2_ and the residual nickel and cobalt oxides occurred at 685–880°C.

Kinetic analysis of the non-isothermal reduction experiments using the iso-conversional method demonstrates increasing activation energy with the progression of the reduction. The activation energy increased from 37.64 kJ/mol (*α* = 0.2) to 98.43 kJ/mol (*α* = 0.9) using the FWO model.

During the isothermal reduction, the average oxygen content in the alloy phases was the lowest at 800°C, and the content of nickel and cobalt in the oxide phases gradually decreased with the increase in reduction temperature. The apparent activation energy for the isothermal tests in the temperature range of 500–700°C was 84.86 kJ/mol, and the reaction process conforms to the 3D diffusion model, implying the rate-controlling step was the inward diffusion of hydrogen within each particle. The apparent activation energy for the isothermal tests in the temperature range of 700–900°C was 51.82 kJ/mol, and the reaction conforms to the R2 contracting area model, suggesting a downward progression of the reduction through the material bed.

## Data Availability

The original contributions presented in the study are included in the article/Supplementary Material, further inquiries can be directed to the corresponding author.
