# Supplementary material for: Hydrogen reduction of spent lithium-ion battery cathode material for metal recovery: Mechanism and kinetics
Source: Front Chem. 2022 Sep 26;10:1019493. doi: 10.3389/fchem.2022.1019493 (PMC9548595; doi:10.3389/fchem.2022.1019493)
Supplement: Supplementary file 1 [file DataSheet1.docx]

***Supporting information***

**Hydrogen reduction of spent lithium-ion battery cathode material for metal recovery: mechanism and kinetics**

Zhu Huang^a,b^, Dawei Yu^a,b,*^, Brian Makuza^a,b^, Qinghua Tian^a,b^, Xueyi Guo^a,b^, Kun Zhang^c^

*^a^ School of Metallurgy and Environment, Central South University, Changsha, Hunan 410083, China*

*^b^ National and Regional Joint Engineering Research Center of Nonferrous Metal Resource Recycling, Changsha, Hunan 410083, China*

*^c^ National WEEE Recycling Engineering Research Centre, Jingmen, Hubei 448124, China*

**Corresponding author*: Dawei Yu

*Email: dawei.yu@csu.edu.cn*

*Tel: (+86)0731-88876255*


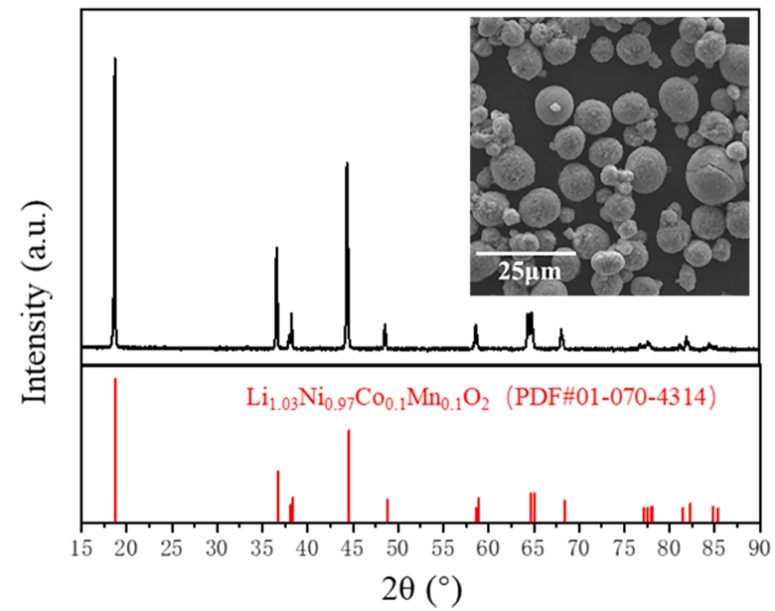


**FIGURE S1**. X-ray diffractogram and SEM micrograph of the untreated cathode powders.


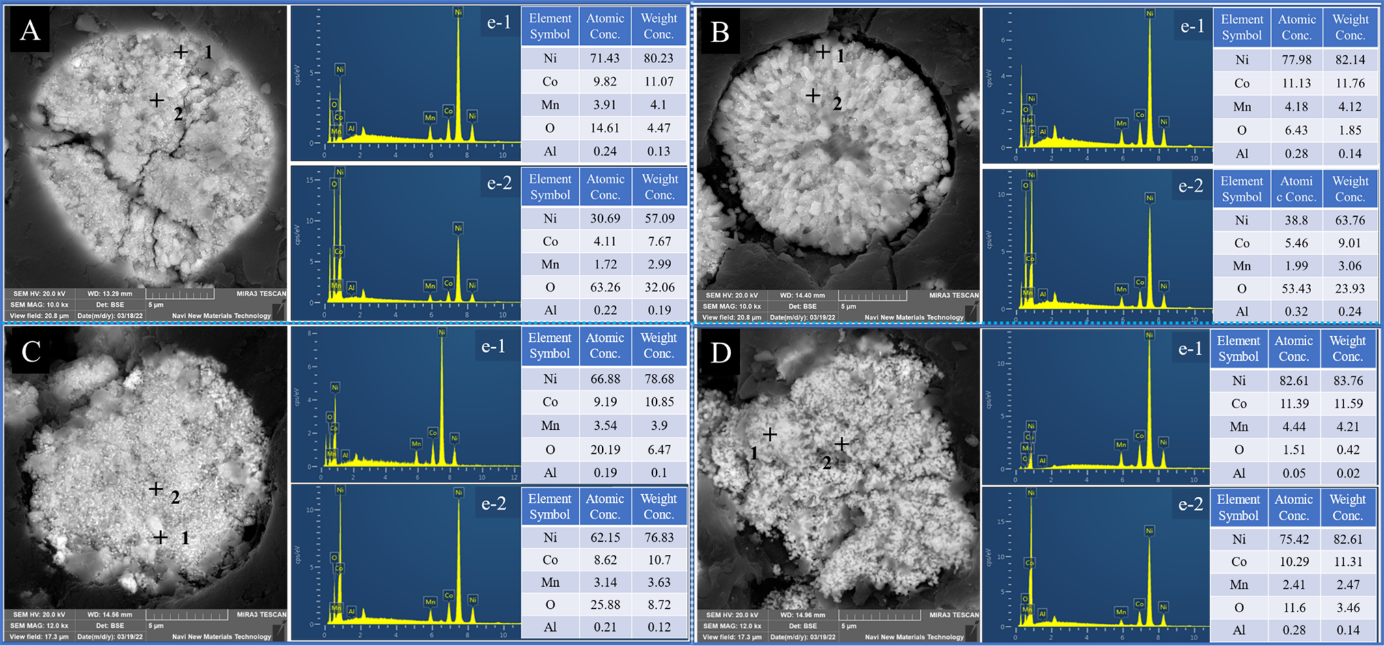


**FIGURE S2**. SEM images and EDS spectra of the reduction products at intermediate reduction temperatures under non-isothermal reduction conditions. A) 420 ℃; B) 450 ℃; C) 475 ℃; D) 553 ℃.


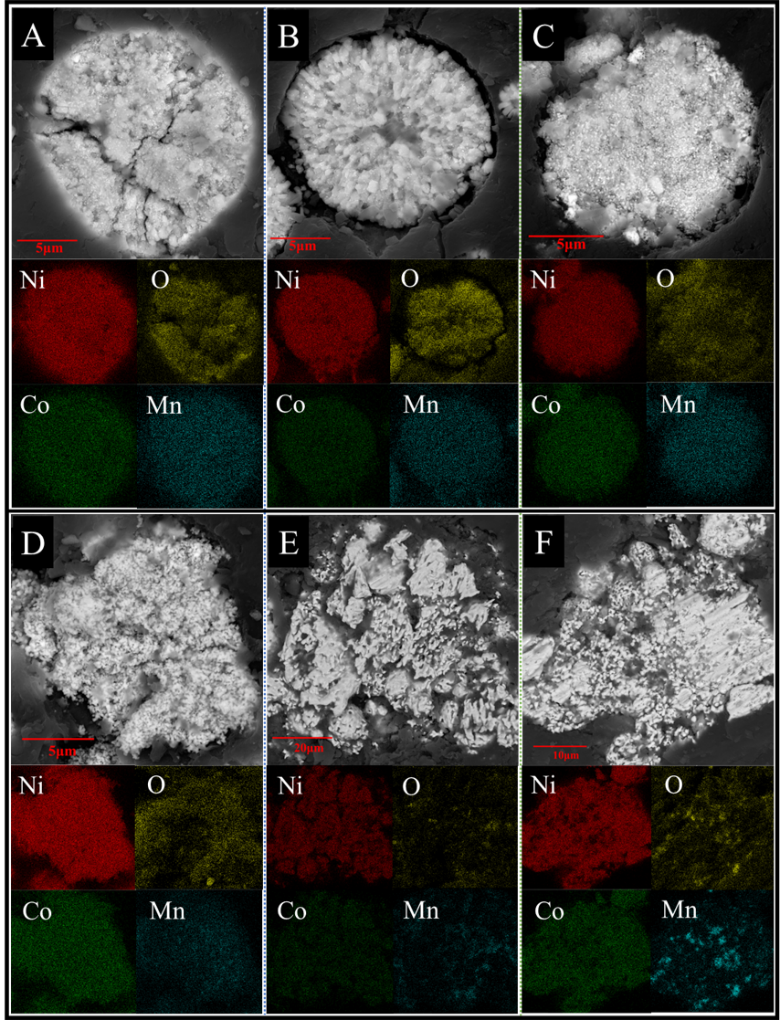


**FIGURE S3**. SEM and elemental mapping images of the reduction products at intermediate reduction temperatures under non-isothermal conditions A) 420 °C;B) 450 °C; C) 475 °C; D) 553 °C; E) 685 °C; F) 880 °C.


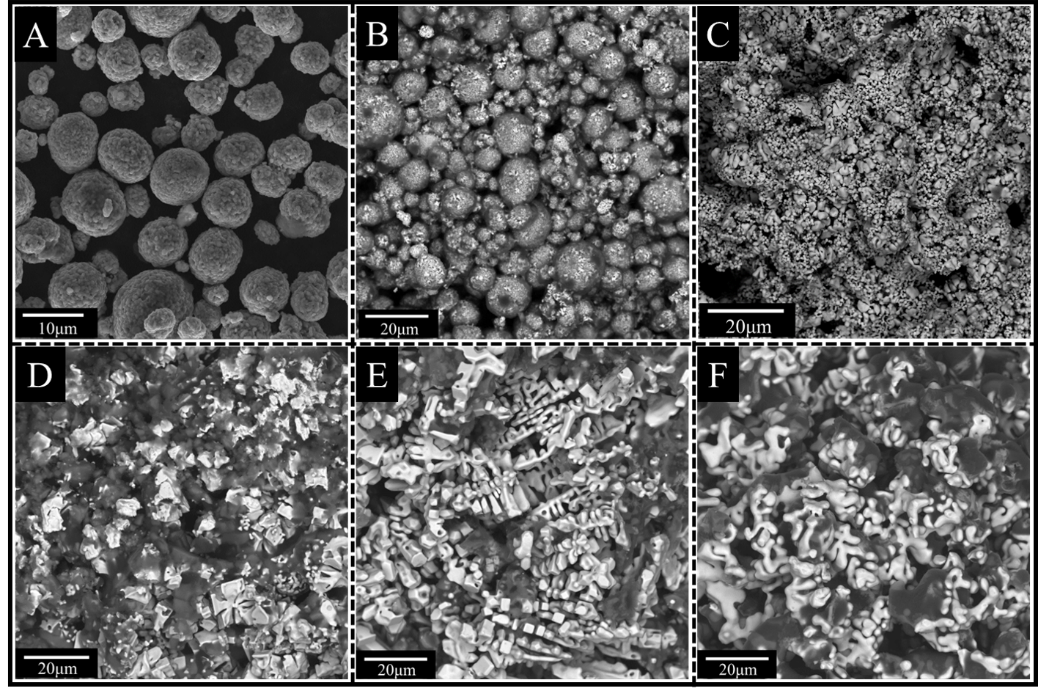


**FIGURE S4**. SEM images (BSE) of the reduction products at different reduction temperatures under isothermal reduction conditions. A) raw material; B) 500 °C; C) 600 °C; D) 700 °C; E) 800 °C; F) 900 °C.


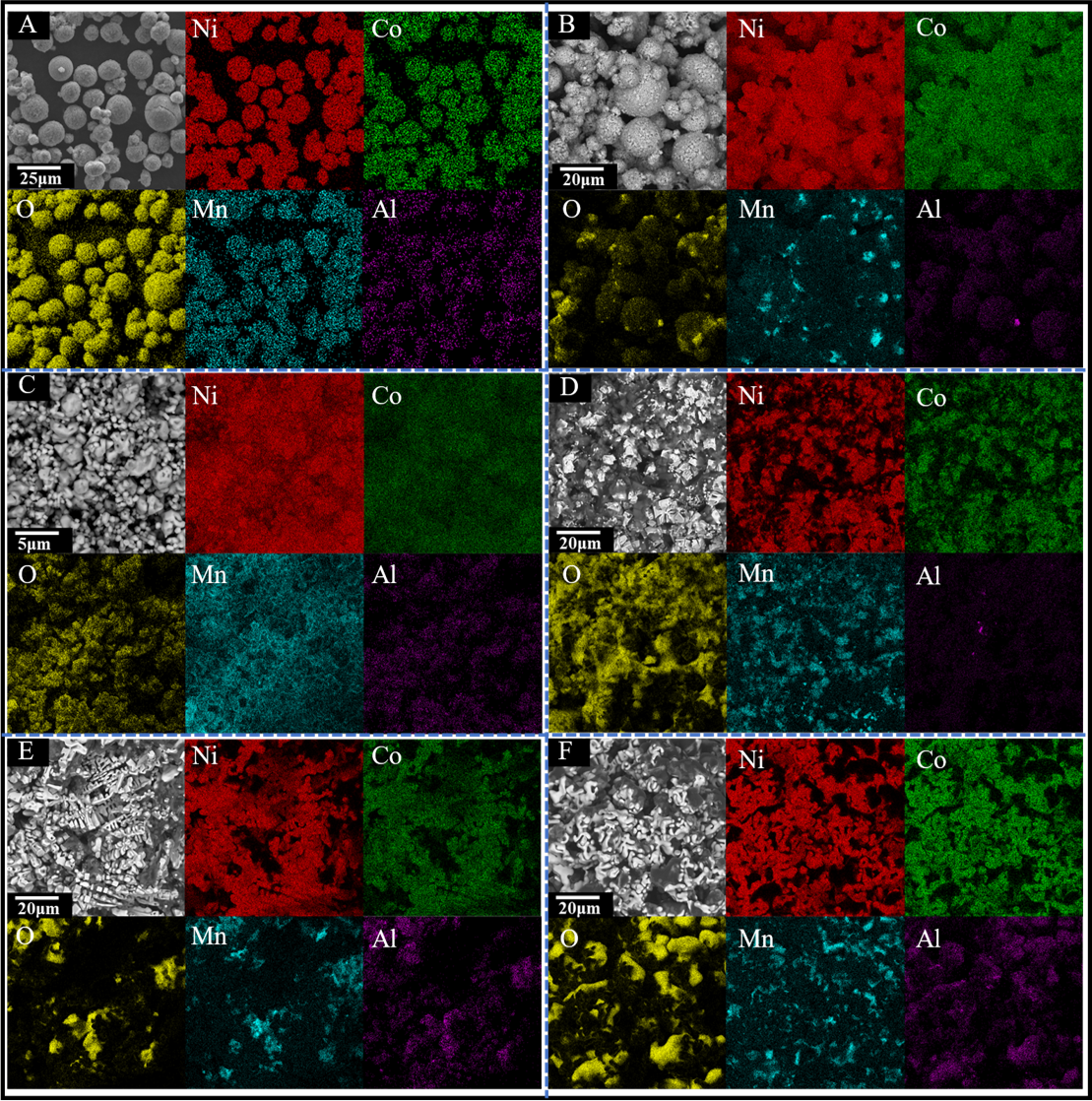


**FIGURE S5**. SEM and elemental mapping images of the reduction product at different temperatures under isothermal reduction conditions A) raw material; B) 500 °C; C) 600 °C; D) 700 °C; E) 800 °C; F) 900 °C.


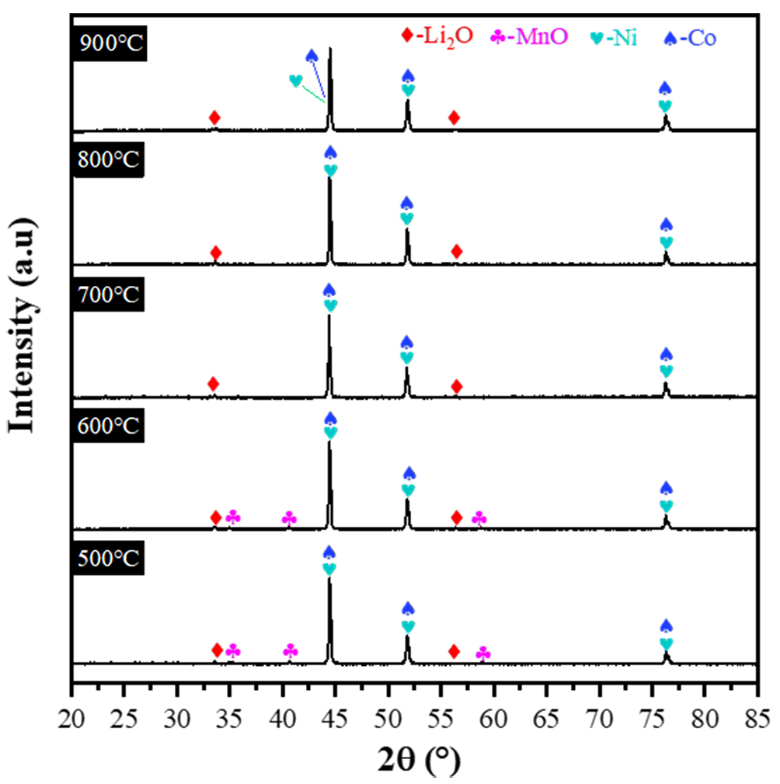


**FIGURE S6**. XRD patterns of the reduction product at different temperatures under isothermal reduction conditions.


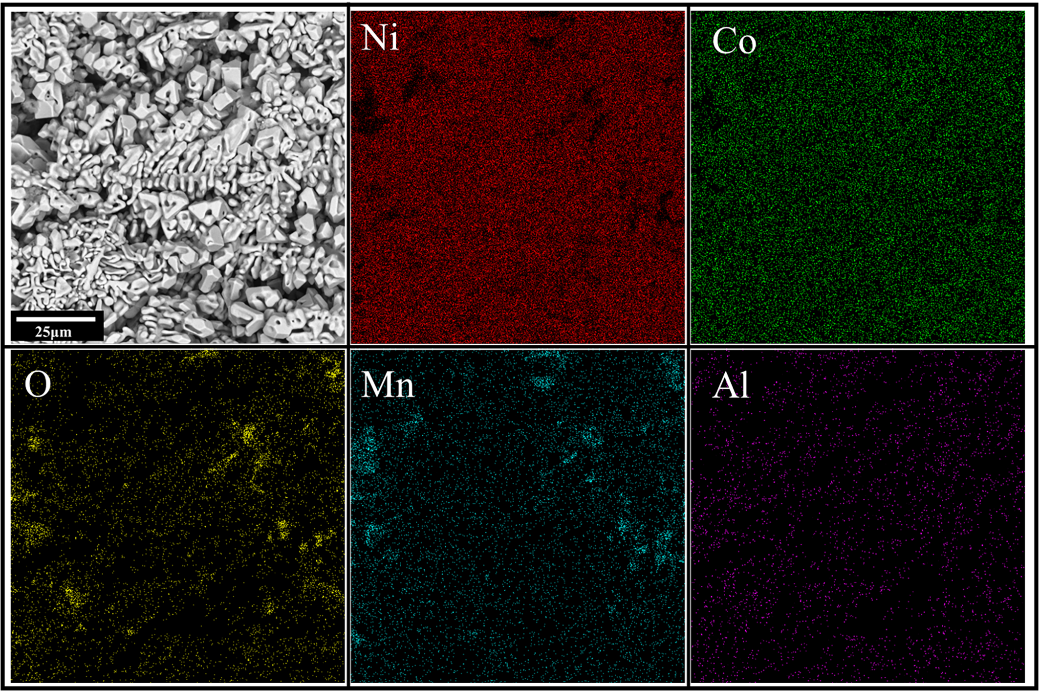


**FIGURE S7**. SEM and elemental mapping images of the residue obtained after water leaching of the reduction product (Reduction temperature 800 ℃, reduction time 3 h).


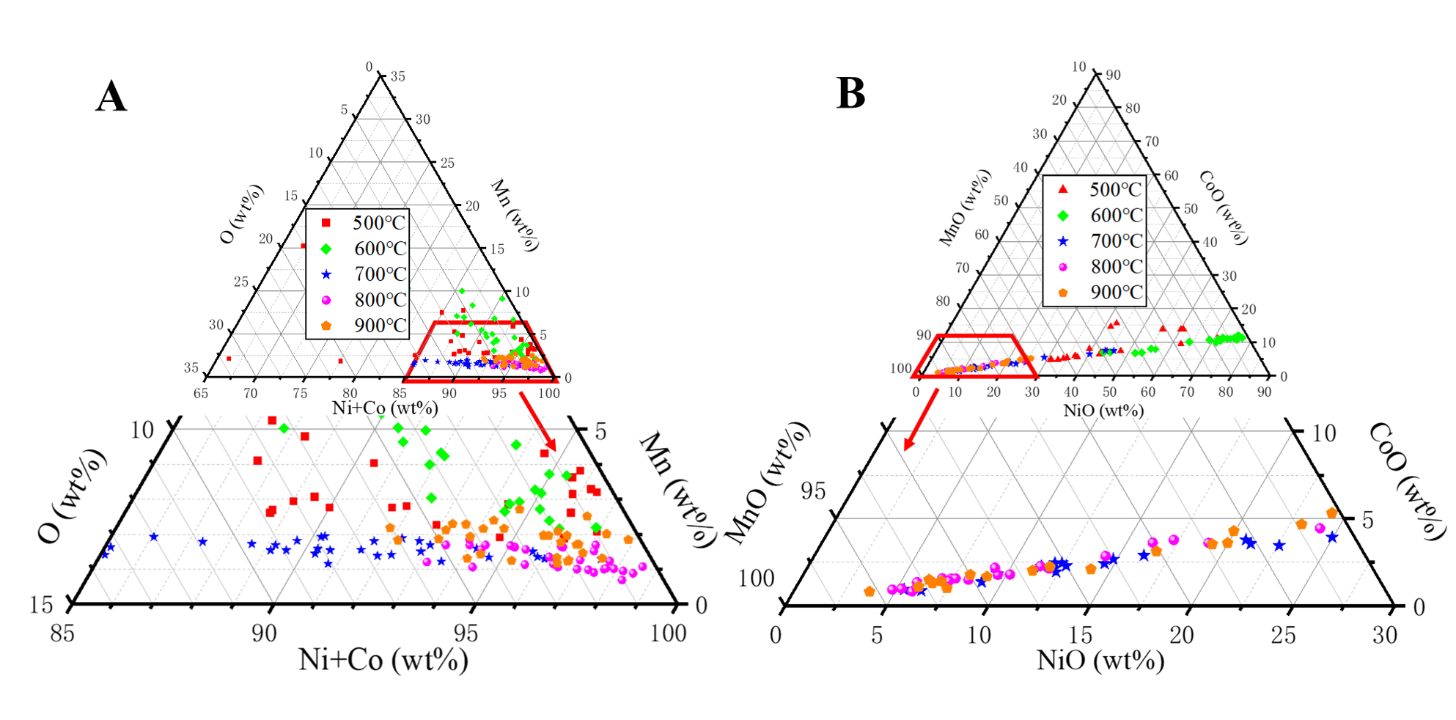


**FIGURE S8.** Ternary plots showing the compositional change of alloys and oxides as a function of hydrogen reduction temperature, analyzed by EDS point analysis: A) Normalized content of Ni+Co, Mn, and O in the alloy phases found in the magnetic fraction; B) Normalized content of NiO, CoO, and MnO found in the oxide phases in the non-magnetic fraction (Assuming the oxides of Ni, Co, and Mn are all in their bivalent state in the non-magnetic fraction).


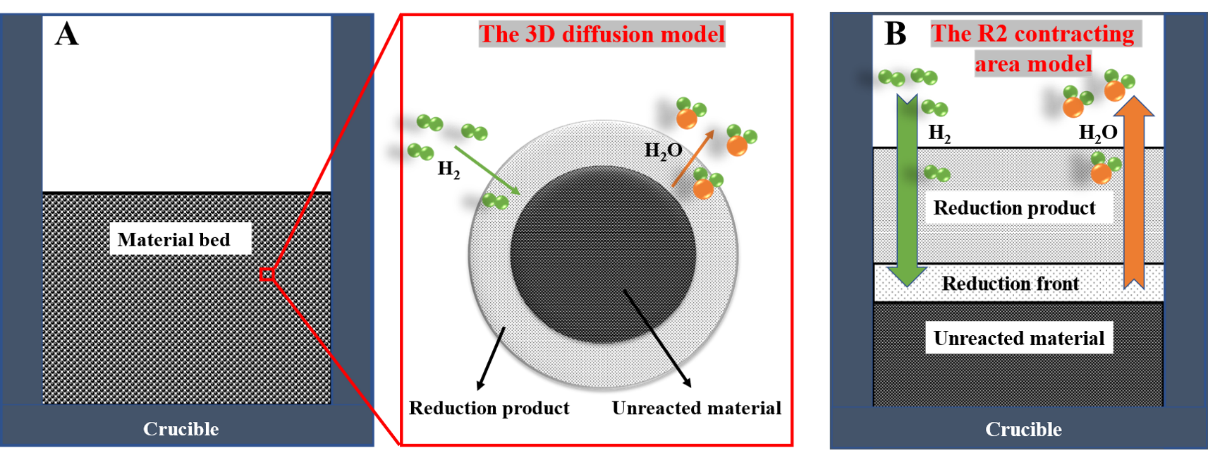


**FIGURE S9.** Schematic representation of the kinetic models A) The 3D diffusion model; B) The R2 contracting area model.
